# Evaluation of stretched penile length (SPL), postnatal penile growth evolution, and micropenis in Brazilian preterm newborns

**DOI:** 10.1016/j.jped.2025.101437

**Published:** 2025-08-27

**Authors:** Bárbara Reis Krämmer, Rita C. Silveira, Eduardo Correa Costa, Matheus Lourenço Mendes, Renato S. Procianoy, Guilherme Guaragna Filho

**Affiliations:** a Pediatric Endocrinologist, Post-graduate Program in Child and Adolescent Health, Faculty of Medicine, Universidade Federal do Rio Grande do Sul (UFRGS), Porto Alegre, Brazil; b Department of Pediatrics, UFRGS, Post-graduate Program in Child and Adolescent Health, Faculty of Medicine, Universidade Federal do Rio Grande do Sul (UFRGS), Porto Alegre, Brazil; c Pediatric Surgeon and Pediatric Urologist, Department of Pediatric Surgery, Hospital de Clínicas de Porto Alegre, Porto Alegre, Brazil; d Pediatric Endocrinology Resident, Department of Endocrinology, Hospital de Clínicas de Porto Alegre, Porto Alegre, Brazil

**Keywords:** Anthropometry, Premature infant, Male genital disease

## Abstract

**Objective:**

to develop a reference curve for SPL in Brazilian preterm newborns, assessing their postnatal growth adjusted for corrected gestational age (cGA).

**Materials and Methods:**

This is a prospective cohort study conducted in southern Brazil. Newborns who were born below 37 weeks of gestational age were selected at tertiary care hospital during the first 3 days of life were selected over a one-year period. SPL was measured weekly until the participants reached 37 weeks of cGA or were discharged from the hospital. Statistical analysis included the Lambda Mu Sigma (LMS) method for growth curve construction and the Bland-Altman test to assess measurement agreement.

**Results:**

A total of 290 SPL measurements were collected from 140 participants. Reference curves were developed for cGA between 26 and 37 weeks. Bland-Altman analysis suggested agreement between measurements taken immediately after birth and those taken during follow-up, inferring that preterm newborns may exhibit penile growth patterns similar to those exhibited during the intrauterine period.

**Conclusion:**

hypothalamic-pituitary-gonadal axis integrity appears to be the primary determinant of post-natal penile growth. The proposed reference curves aid in the early diagnosis of hormonal and genetic alterations, allowing for timely medical interventions.

## Introduction

The assessment of the external genitalia is an essential component of the newborn's clinical examination, and the measurement of penile length provides valuable insights for the diagnosis of conditions such as Disorders of Sexual Development (DSD), Hypogonadotropic Hypogonadism, and Hypopituitarism [[1], [2], [3], [4]].

Micropenis is defined as a structurally normal penis with a SPL measurement bellow 2.5 standard deviations (SD) of the mean. Thus, the diagnosis of micropenis relies on the comparison of the child’s SPL with stablished reference values, which are scarce for preterm newborns and nonexistent for the Brazilian population [5]. SPL may vary across different populations, in this way, the establishment of local standard parameters remains a relevant topic [1,4,6].

It is estimated that 11% of newborns are born preterm, i.e., before 37 weeks of gestational age (GA) [7] In absolute numbers, Brazil ranks 10th in the number of preterm births, with 279,000 newborns having a GA below 37 weeks in 2010 [7].

This study aims to assess SPL in preterm newborns, to develop reference curves for penile size in Brazilian children, and to evaluate the postnatal penile growth pattern in a cohort of preterm newborns adjusted for cGA.

## Materials and methods

This is a prospective, single-center cohort study conducted at a tertiary hospital in southern Brazil. The sample was obtained through consecutive selection of preterm newborns who began follow-up within three days of life, between September 2023 and September 2024.

Newborns with a GA of less than 37 weeks at birth, defined by ultrasound dating before 22 weeks of GA, were recruited from the Neonatology Service at the Hospital de Clínicas de Porto Alegre (HCPA) [8]., The sample size was estimated at approximately 61 individuals, considering a 95% confidence level, a standard deviation of 3.97 mm, according to the systematic review by López-Soto et al. (2021) on penile length at birth, and an acceptable standard error of 1 mm. Informed consent was obtained from the parents during neonatal hospitalization. The study was submitted and approved by the HCPA Research Ethics Committee (CAAE 70073623.6.0000.5327). Patients with clinical instability, evident genital alterations (e.g., ambiguous genitalia, hypospadias, late preterm boys with cryptorchidism), genetic syndromes, or major midline defects (e.g., congenital heart disease, gastroschisis, omphalocele, cleft lip/palate, and congenital nervous system malformations) were excluded from the study. In contrast, children without a predicted early discharge date—allowing for the possibility of testicular descent being observed—along with preterm infants under 34 weeks with cryptorchidism, were included.

Selected patients underwent SPL and penile diameter (PD) measurements within the first three days of life in a warm temperature-controlled environment. SPL was measured under maximum traction from the pubopenile cutaneous angle to the glans tip, with depression of the prepubic fat, using a ruler placed dorsally along the penis, as described by Schonfeld and Beebe [9]. The same experienced examiner performed all standardized measurements three times, with the mean value recorded. Measurements were repeated weekly until the infant reached 37 weeks of cGA or was discharged from the hospital. PD was measured once using a Luatek LWJ308 digital caliper with millimeter precision (0.2 mm accuracy, 0.1 mm resolution). Clinical assessment was not performed during periods of clinical instability, and missing data were excluded from analysis. The intraclass correlation coefficient was used to assess the consistency among the three measurements, which were subsequently averaged.

Clinical data were collected from medical records, including maternal and gestational comorbidities, neonatal complications, and additional anthropometric data. Maternal smoking status was self-reported, while psychoactive substance use was detected through universal screening tests conducted that are de standard at the maternity hospital the study was conducted. Small for gestational age was defined by length or weight at birth bellow -2 Z scores using Intergrowth 21st calculator available at the https://intergrowth21.ndog.ox.ac.uk website. Large for Gestational Age was defined by length or weight at birth above 2 Z scores using the same calculator. Hypoglycemia was defined as a point-of-care capillary blood glucose below 47 mg/dL, as defined by the American Academy of Pediatrics. Micropenis was defined as a SPL of less than -2.5 DP [10].

Parametric variables were described as mean and SD, while non-parametric variables were described as median and interquartile range. The Shapiro-Wilk and Kolmogorov-Smirnov were used to assess normality. The Bland-Altman analysis compared SPL measurements taken within the first three days of life with those obtained during follow-up, comparing newborns with similar CGAs immediately after birth with those who remained in the study.

SPL reference curves were developed using the Lambda Mu Sigma (LMS) method, a recognized method used for the elaboration of growth curves by the WHO and CDC since its development in 1992 [11,12]. This method summarizes data using three parametric curves: the L curve captures distribution skewness, the M curve represents the central tendency, and the S curve describes the coefficient of variation. The Box-Cox transformation corrects data skewness, and curves are smoothed by penalized maximum likelihood [[11], [12], [13]]. We elaborated the reference curves using R language version 4.4.2 and the GAMLSS Library version 5.4-22 (additional information of the model in the supplementary material S1).

Categorical and continuous variables analysis were evaluated using SPSS version 22.0. Bland-Altman tests performance were performed with R language version 4.4.2.

## Results

A total of 140 participants were selected (Table 1). Of these, 86 had early discharge and had only one SPL measurement recorded within the first 72 hours of life. The remaining 56 patients remained hospitalized for more than a week and underwent follow-up, with a median of two observations per patient (ranging from one to 11 observations). We had a total of 312 weeks of follow-up, during which 290 SPL measurements were obtained - 22 measurements were not performed due to clinical instability. The intraclass correlation coefficient among the three measurements was 0.937 (p < 0.0001).

**Table 1:** Population characteristics.

			**n (% of total)**	**Mean (SD)**	**Median (IQR)**
cGA at first evaluation		24 25 26 27 28 29 30 31 32 33 34 35 36 37	1 (0.7) 0 (0) 3 (2.1) 2 (1.4) 1 (0.7) 1 (0.7) 5 (3.6) 3 (2.1) 6 (4.3) 9 (6.4) 24 (17.1) 17 (12.1) 56 (40) 12 (8.6)	34.4 (2.6)	35 (2)
Self-reported race		White Black Mixed-race (“Pardo”)	105 (75) 16 (11) 19 (13)		
Maternal comorbidities		HTN Pre-eclampsia PAS Smoking HELLP syndrome	30 (21) 18 (12) 13 (9.3) 12 (8.6) 2 (1.4)		
Betamethasone use during gestation			32 (22.9)		
Anthropometry data at birth		SGA AGA LGA	20 (14.3) 116 (82.9) 4 (2.9)		
Twin birth		No Yes	115 (82.1) 25 (17.9)		
Cryptorchidism		First evaluation Final evaluation	15 (10.7) 10 (7.1)		
Apgar	Minute 1 Minute 5	7-10 4-6 0-3 7-10 4-6 0-3	103 (73.6) 28 (20) 9 (6.4) 133 (95) 7 (5) 0 (0)		8 (2) 9 (1)
Neonatal comorbidities		Neonatal Jaundice* MV NE Parenteral nutrition Hypoglycemia** Early-onset NS Late-onset NS AED use PIVH grades 1 e 2 PIVH grades 3 e 3	40 (28,6) 16 (11,4) 0 (0) 23 (16,4) 65 (46,4) 19 (13,6) 11 (7,9) 8 (5,7) 4 (2,9) 1 (0,7)		

*= with phototherapy use. **= first 72 hours of life. AED – Anti Epileptic drug; AGA – Adequate for gestational age; cGA – Corrected Gestational Age; HTN – Previous or Gestational Hypertension; HELLP – Hemolysis, elevated liver enzymes and low blood platelet count; IQR – Interquartile Range; LGA – Large for gestational age; NE – Necrotizing enterocolitis; NS– Neonatal Sepsis; PAS – Psychoactive substances; PIVH – Peri-intraventricular hemorrhage r; SGA– Small for gestational age; VM – Mechanical Ventilation.

Bland-Altman analysis suggested that SPL measurements at similar cGA were comparable (bias = -0.6030167; 95% CI: -1.7117 to 0.5057; agreement limits: -4.023 to 2.817). Therefore, all 290 SPL measurements from the 140 patients were used to construct SPL growth charts for cGA (supplementary material S2).

Bland-Altman analysis suggests that similar cGAs had comparable SPL (bias = -0.6030167 IC 95% 1,7117 e 0,5057; limits of agreement Li – Ls -4,023 a 2,817), when comparing children evaluated during the first 3 days of life and children of equivalent cGAs that had follow-up measurements. Therefore, the 290 SPL measurements from the 140 participants were used to create the SPL chart for cGA.

**Figure 1:**
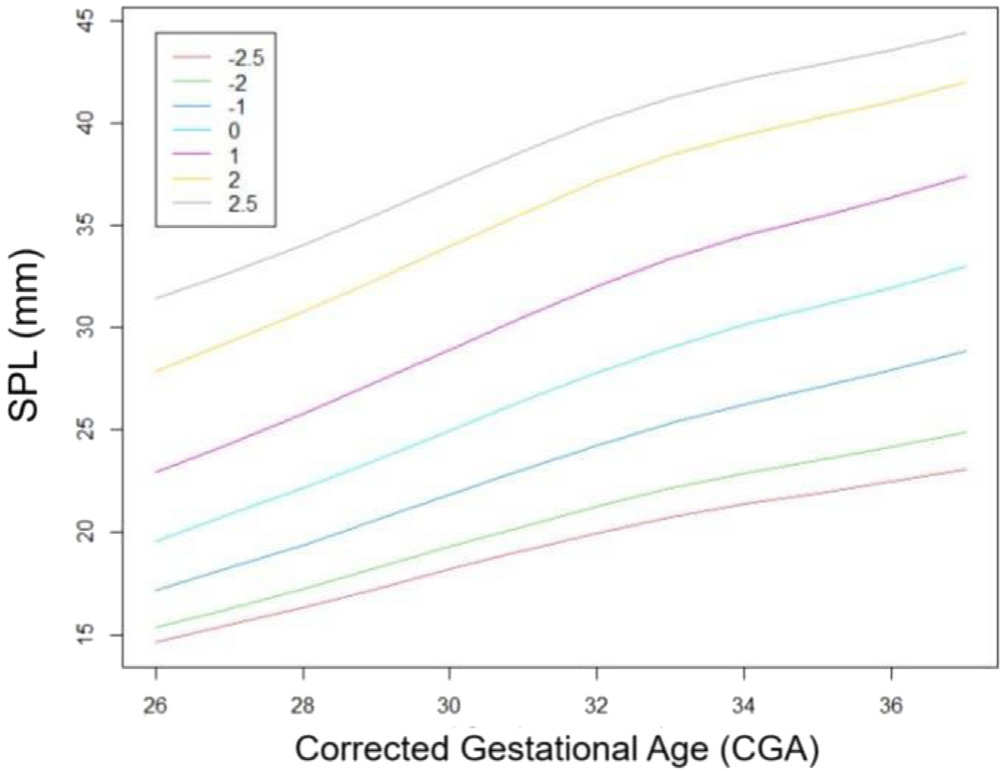
Stretched penile length (SPL) according to corrected gestational age (cGA) in millimeters (mm) Z scores.

The SPL curve elaborated using de LMS method is presented in Figure 1. The proposed values for mean SPL for cGA, macropenis and micropenis are presented in Table 2.

**Table 2:** Micropenis, average size and macropenis by corrected gestational age (cGA) in millimeters (mm)

CGA	Z SCORE -2.5	MEAN	Z SCORE +2.5
26	14.6	19.6	31.5
27	15.5	20.8	32.7
28	16.3	22.2	34
29	17.3	23.5	35.5
30	18.2	25	37.1
31	19.1	26.4	38.6
32	20	27.8	40
33	20.7	29	41.2
34	21.4	30.1	42.1
35	21.9	31	42.8
36	22.5	31.9	43.5
37	23.1	33	44.4

The assessment of PD adjusted for cGA was carried out in 108 patients, from which 215 measurements were obtained. The means and standard deviations of PD by gestational age are represented in Table 3.

**Table 3:** Penile diameter (PD) and standard deviation (SD) by corrected gestational age (cGA) in millimeters (mm)

CGA	MEAN PD (MM)	SD (MM)
26	4.1	0.9
27	5.3	0.4
28	5.1	0.8
29	5.6	1
30	6.1	1
31	6.2	0.8
32	6.8	1
33	7.1	1
34	7.8	1.2
35	8.1	1.2
36	8.4	1.3
37	8.5	0.9

## Discussion

This cohort represents the first anthropometric SPL study in Brazilian preterm newborns. We found no other studies in medical literature evaluating postnatal SPL by corrected age in preterm babies of any nationality.

Factors such as prematurity, primiparity, twin pregnancies, and intrauterine growth restriction are known risk factors for hypospadias and cryptorchidism - therefore, the health of the gonadal axis in preterm newborns should be a matter of interest for both pediatricians and pediatric endocrinologists [14]. Additionally, given the increasing trend of preterm births in Brazil in recent years, particularly during the pandemic years, this topic becomes yet more relevant [15].

The SPL measurements were considered methodologically adequate due to the very strong agreement indicated by the intraclass correlation coefficient. Bland-Altman analysis suggests that preterm infants with similar cGAs are comparable when it comes to SPL, indicating that postnatal penile growth does not appear to be affected by the extrauterine environment. It is reported that mini-puberty in preterm newborns is more prolonged and more intense—gonadotropin and androgen levels are typically higher and persist for longer, with greater testicular and penile growth during this period, which may justify our findings [16,17]. Furthermore, we consider that this positive postnatal evolution of SPL as an indication that the primary determinant of penile growth is the integrity of the hypothalamic-pituitary-gonadal axis.

In newborns born at term, the average SPL is 3 to 3.6 cm, depending on the reference and population studied, and an SPL below 2.3 to 2.5 cm is typically considered micropenis in this age group [4,18,19]. The cut-off values we found in this cohort for SPL in newborns with a cGA of 37 weeks are similar to those reported for term infants.

Normal penile development results from strictly regulated sequential processes controlled by a complex hierarchical network of regulatory genes and proteins, particularly between gestational weeks 8 and 14 [[20], [21], [22]]. Gonadal androgen production is initially stimulated by human chorionic gonadotropin produced in the placenta and, from the second trimester onwards, depends on pituitary stimulation via luteinizing hormone [17,23]. Therefore, SPL reflects not only placental health but also proper central nervous system formation, as well as the child's genetic, hormonal, and gonadal integrity.

In our study, the presence of modifiable gestational risk factors such as smoking and psychoactive substance (PAS) use (cannabis and cocaine) was alarming —8.6% and 9.3%, respectively. The prevalence of PAS use among pregnant women in Brazil is unknown, while smoking rates in our population, although high, were lower than those reported in previous studies [24,25]. Maternal smoking during pregnancy may have deleterious effects on gonadal function in adulthood, with permanent negative impacts on spermatogenesis [14]. The potential effects of prenatal PAS exposure on male reproductive health remain partially understood. In animal models, prenatal cocaine exposure has been associated with reduced anogenital distance, a marker of gonadal health similar to SPL [26]. Cannabis use and cannabis use during pregnancy, are on the rise, and while its effects on male genital development are not fully known, THC exposure in animal models has been linked to adverse impacts on spermatogenesis and testicular function [27].

Our main limitations were the asymmetric representation of late preterm infants compared to moderate and extreme preterm infants, as well as the low representation of children born SGA/with very low birth weight, which limited comparisons of SPL with those born with an adequate weight for gestational age (AGA). This is partly due to the single-center nature of the study and partly due to the frequent clinical instability in the extreme preterm population, which prevents physical examinations in the first 72 hours of life. Multicenter studies or longer follow-up periods are necessary to address this gap. Due to this limitation of the study, the cut off suggestions for micropenis of extreme preterm infants should be interpreted cautiously.

Another limitation was the lack of anogenital distance and testicular volume assessments [28]. However, given that the study included extreme preterm infants, we dealt with a particularly sensitive population, making excessive handling during physical examination a concern.

Additionally, we must address that most measurements included were of children with limited follow-up and inferences about growth trends may be limited in generalizability. Our primary goal was to propose a reference curve for SPL length in preterm infants, with an exploratory aim to observe postnatal SPL patterns of growth. Further studies with larger sample sizes or extended follow-up after hospital discharge are needed to address this gap.

Micropenis is a clinical sign of hypogonadotropic hypogonadism, isolated growth hormone deficiency, hypopituitarism, genetic syndromes, disorders of sex differentiation, hormonal synthesis and androgen action disorders, luteinizing hormone receptor defects, and fetal exposure to endocrine disruptors [29,30]. The use of specific population-based reference curves is essential for the comprehensive care of preterm newborns and the early diagnosis of conditions whose long-term outcomes depend on timely medical intervention.

## Conclusions

The development of reference curves for SPL in Brazilian preterm newborns is a positive step towards comprehensive care in neonatology. This is the first study of anthropometric evaluation of SPL in Brazilian preterm newborns as well as the first cohort to assess postnatal SPL measurements by corrected gestational age during the neonatal period.

Our results suggest that the integrity of the hypothalamic-pituitary-gonadal axis is the main determinant of the development of the external male genitalia. The correlation between postnatal penile growth and the measurement of variables such as serum androgens could be a next step to further clarify this finding, along with the evaluation of clinical variables such as testicular volume and anogenital distance.

The proposed reference curve is a practical tool for the early diagnosis of micropenis and DSDs in preterm newborns. The identification of conditions that require early intervention provides a better prognosis and improves the quality of life of affected patients.

## Author contributions

Bárbara Reis Krämmer: Conceptualization, Data curation, Formal analysis, Investigation, Writing - original draft.

Rita C. Silveira: Formal analysis, Supervision, Validation.

Eduardo Correa Costa: Methodology, Visualization, Writing - original draft.

Matheus Lourenço Mendes: Data curation.

Renato S. Procianoy: Methodology, Supervision, Validation.

Guilherme Guaragna Filho: Contributed to the formulation of the research question and study objectives; participated in methodology design; supervised clinical data collection; revised the manuscript and critically reviewed its intellectual content. Conceptualization, Data curation, Formal analysis, Methodology, Supervision, Writing - original draft, Writing - review & editing.

## Institutions

Post-graduate Program in Child and Adolescent Health. Faculty of Medicine. Universiade Federal do Rio Grande do Sul. Hospital de Clínicas de Porto Alegre

## Funding

The study was conducted with support from the Fundo de Incentivo à Pesquisa (FIPE) of the Hospital de Clínicas de Porto Alegre where it was carried out.

## Conflicts of interest

The authors declare no conflicts of interest.

## Appendix Supplementary materials

Image, application 1
