# Supplementary material for: Evaluation of stretched penile length (SPL), postnatal penile growth evolution, and micropenis in Brazilian preterm newborns
Source: J Pediatr (Rio J). 2025 Aug 27;101(6):101437. doi: 10.1016/j.jped.2025.101437 (PMC12409386; doi:10.1016/j.jped.2025.101437)
Supplement: Supplementary file 1 [file mmc1.docx]

**Supplementary material 1 - LMS model**

**library**(haven)

*#setwd("G:/.shortcut-targets-by-id/1aQQsgqJwFMNWcPtDXER-hYL_dbLOSXke/Assessorias/Assessorias Gustavo/Barbara BK 200620")*

bd <- read_sav("Database completo - 140 pacientes.sav")

dim(bd)

## [1] 312 71

*# names(bd)*

bd=bd[,c("IGCsem","OrdemMedidasPaciente","CPT","Pig")]

dim(bd)

## [1] 312 4

head(bd)

## # A tibble: 6 x 4

## IGCsem OrdemMedidasPaciente CPT Pig

## <dbl> <dbl> <dbl> <dbl>

## 1 37 6 32 0

## 2 35 8 32 0

## 3 33 6 32 0

## 4 32 5 38 0

## 5 34 4 32 0

## 6 35 5 34 0

**library**(gamlss)

## Warning: pacote 'gamlss' foi compilado no R versão 4.4.2

## Carregando pacotes exigidos: splines

## Carregando pacotes exigidos: gamlss.data

##

## Anexando pacote: 'gamlss.data'

## O seguinte objeto é mascarado por 'package:datasets':

##

## sleep

## Carregando pacotes exigidos: gamlss.dist

## Warning: pacote 'gamlss.dist' foi compilado no R versão 4.4.2

## Carregando pacotes exigidos: nlme

##

## Anexando pacote: 'nlme'

## O seguinte objeto é mascarado por 'package:dplyr':

##

## collapse

## Carregando pacotes exigidos: parallel

## ********** GAMLSS Version 5.4-22 **********

## For more on GAMLSS look at https://www.gamlss.com/

## Type gamlssNews() to see new features/changes/bug fixes.

plot(bd$CPT~bd$IGCsem)


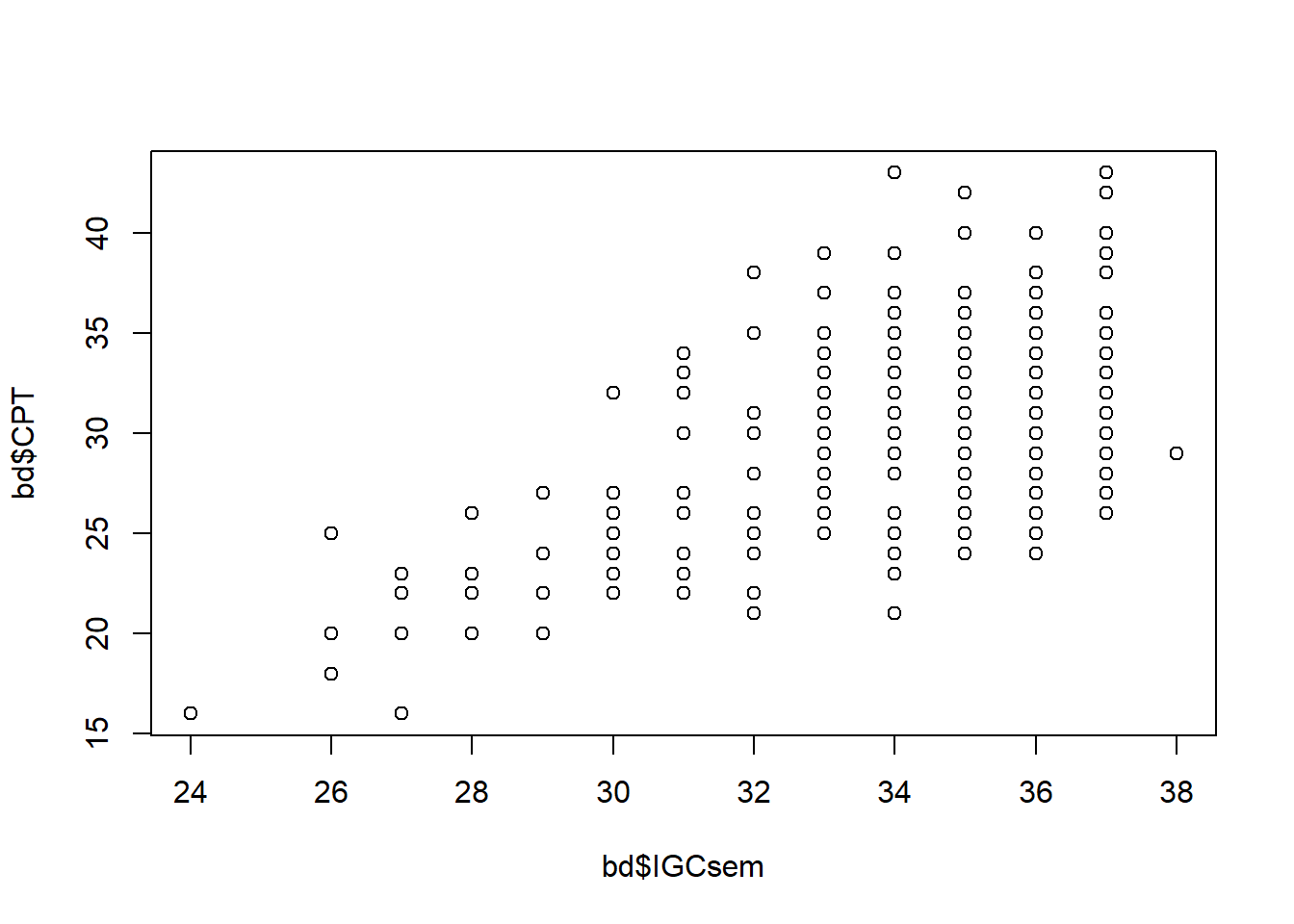


bd = subset(bd,IGCsem>=26&IGCsem<38)

plot(bd$CPT~bd$IGCsem)


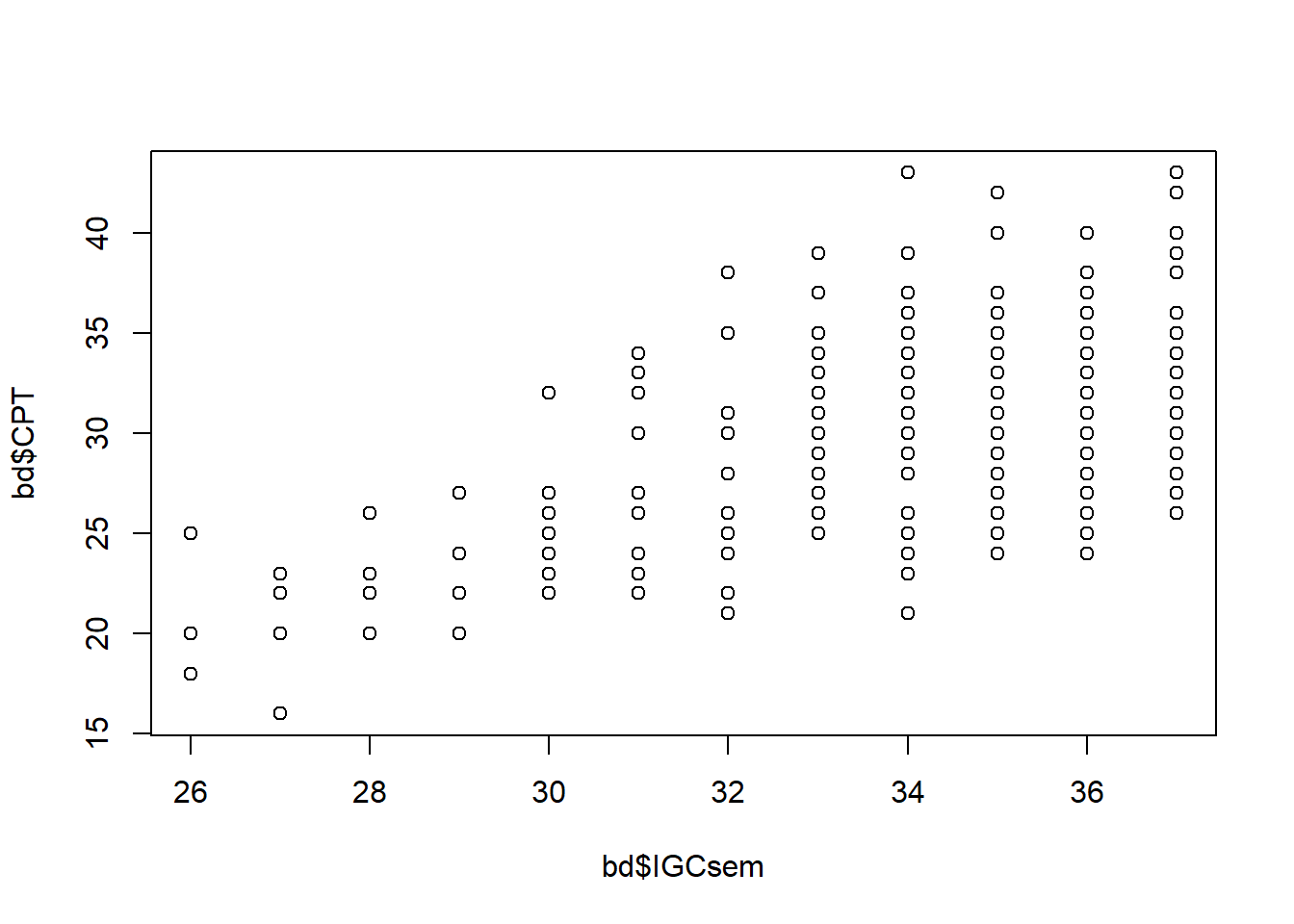


dim(bd)

## [1] 309 4

bd=na.omit(bd)

dim(bd)

## [1] 288 4

m0=lms(CPT,IGCsem,data=bd,trans.x=F,k=2)*#k=2 is the penalty used in the GAIC=BIC*

## *** Initial fit***

## GAMLSS-RS iteration 1: Global Deviance = 1629.278

## GAMLSS-RS iteration 2: Global Deviance = 1629.278

## *** Fitting BCCGo ***

## GAMLSS-RS iteration 1: Global Deviance = 1618.579

## GAMLSS-RS iteration 2: Global Deviance = 1618.094

## GAMLSS-RS iteration 3: Global Deviance = 1618.239

## GAMLSS-RS iteration 4: Global Deviance = 1618.284

## GAMLSS-RS iteration 5: Global Deviance = 1618.294

## GAMLSS-RS iteration 6: Global Deviance = 1618.297

## GAMLSS-RS iteration 7: Global Deviance = 1618.298

## GAMLSS-RS iteration 8: Global Deviance = 1618.298

## *** Fitting BCPEo ***

## GAMLSS-RS iteration 1: Global Deviance = 1617.905

## GAMLSS-RS iteration 2: Global Deviance = 1616.224

## GAMLSS-RS iteration 3: Global Deviance = 1616.02

## GAMLSS-RS iteration 4: Global Deviance = 1615.949

## GAMLSS-RS iteration 5: Global Deviance = 1615.899

## GAMLSS-RS iteration 6: Global Deviance = 1615.862

## GAMLSS-RS iteration 7: Global Deviance = 1615.841

## GAMLSS-RS iteration 8: Global Deviance = 1615.825

## GAMLSS-RS iteration 9: Global Deviance = 1615.818

## GAMLSS-RS iteration 10: Global Deviance = 1615.811

## GAMLSS-RS iteration 11: Global Deviance = 1615.806

## GAMLSS-RS iteration 12: Global Deviance = 1615.801

## GAMLSS-RS iteration 13: Global Deviance = 1615.797

## GAMLSS-RS iteration 14: Global Deviance = 1615.793

## GAMLSS-RS iteration 15: Global Deviance = 1615.79

## GAMLSS-RS iteration 16: Global Deviance = 1615.787

## GAMLSS-RS iteration 17: Global Deviance = 1615.784

## GAMLSS-RS iteration 18: Global Deviance = 1615.782

## GAMLSS-RS iteration 19: Global Deviance = 1615.78

## GAMLSS-RS iteration 20: Global Deviance = 1615.779

## Warning in RS(): Algorithm RS has not yet converged

## *** Fitting BCTo ***

## GAMLSS-RS iteration 1: Global Deviance = 1621.109

## Warning in additive.fit(x = X, y = wv, w = wt * w, s = s, who = who,

## smooth.frame, : additive.fit convergence not obtained in 30 iterations

## GAMLSS-RS iteration 2: Global Deviance = 1618.298

## GAMLSS-RS iteration 3: Global Deviance = 1618.283

## GAMLSS-RS iteration 4: Global Deviance = 1618.282


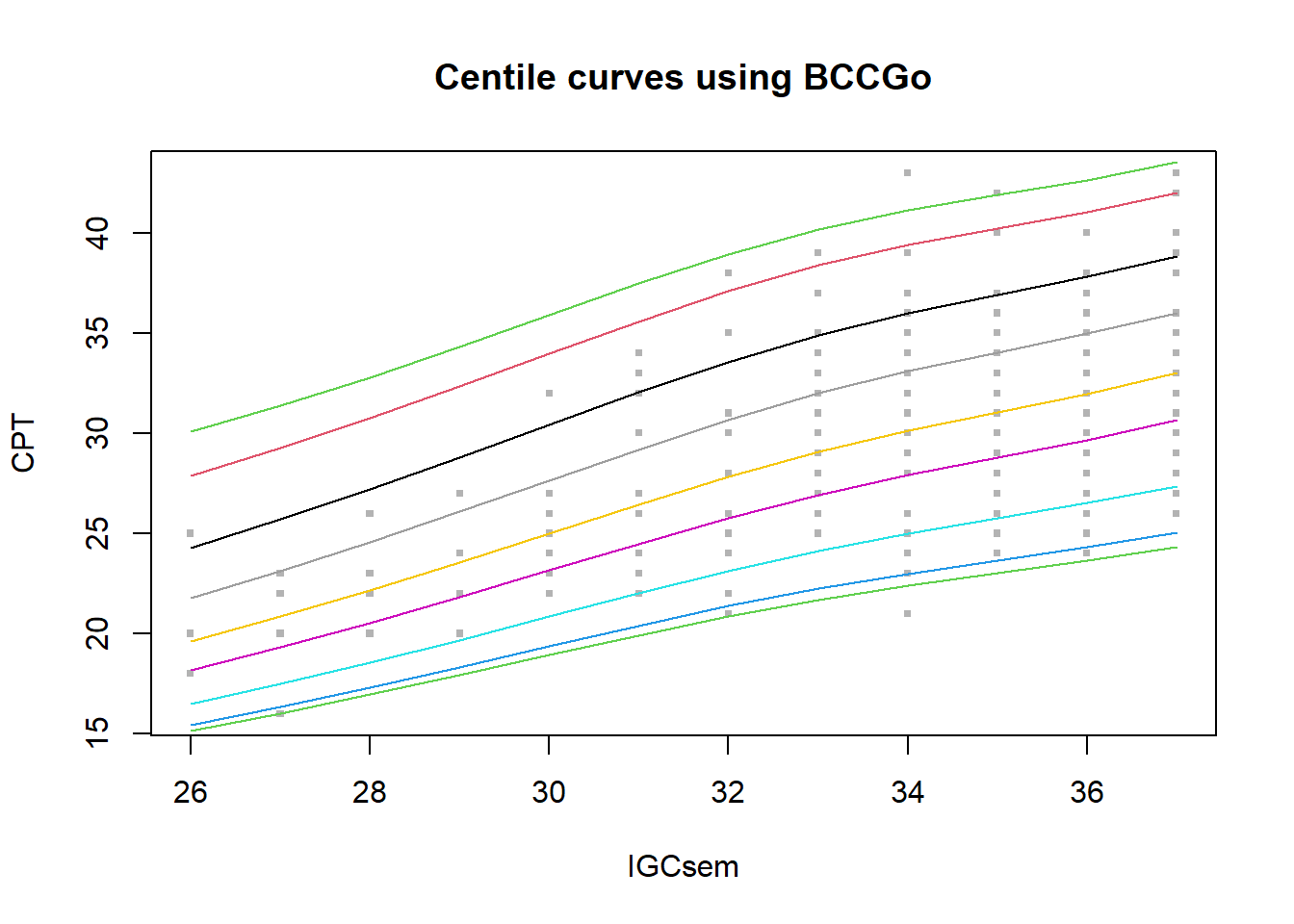


## target calib. sample

## 0.4% 0.4 1.54 0.694

## 2% 2.0 2.45 2.431

## 10% 10.0 8.60 9.375

## 25% 25.0 29.04 25.000

## 50% 50.0 50.26 50.000

## 75% 75.0 75.82 75.347

## 90% 90.0 90.73 90.278

## 98% 98.0 97.72 97.222

## 99.6% 99.6 98.99 99.306

## Warning in regularize.values(x, y, ties, missing(ties)): colapsando para

## valores de 'x' únicos

## Warning in regularize.values(x, y, ties, missing(ties)): colapsando para

## valores de 'x' únicos

## Warning in regularize.values(x, y, ties, missing(ties)): colapsando para

## valores de 'x' únicos

m0$family

## [1] "BCCGo" "Box-Cox-Cole-Green-orig."

edfAll(m0)

## $mu

## $mu$`pb(x, df = mu.df)`

## [1] 3.437019

##

##

## $sigma

## $sigma$`pb(x, df = sigma.df)`

## [1] 2.202436

##

##

## $nu

## $nu$`pb(x, df = nu.df)`

## [1] 2.000006

paste0("Os parametros da distribuição ",m0$family," estimados foram mu=",edfAll(m0)$mu," sigma=",edfAll(m0)$sigma," nu=",edfAll(m0)$nu)

## [1] "Os parametros da distribuição BCCGo estimados foram mu=3.43701923528358 sigma=2.20243597893596 nu=2.0000059875783"

## [2] "Os parametros da distribuição Box-Cox-Cole-Green-orig. estimados foram mu=3.43701923528358 sigma=2.20243597893596 nu=2.0000059875783

plot(m0)


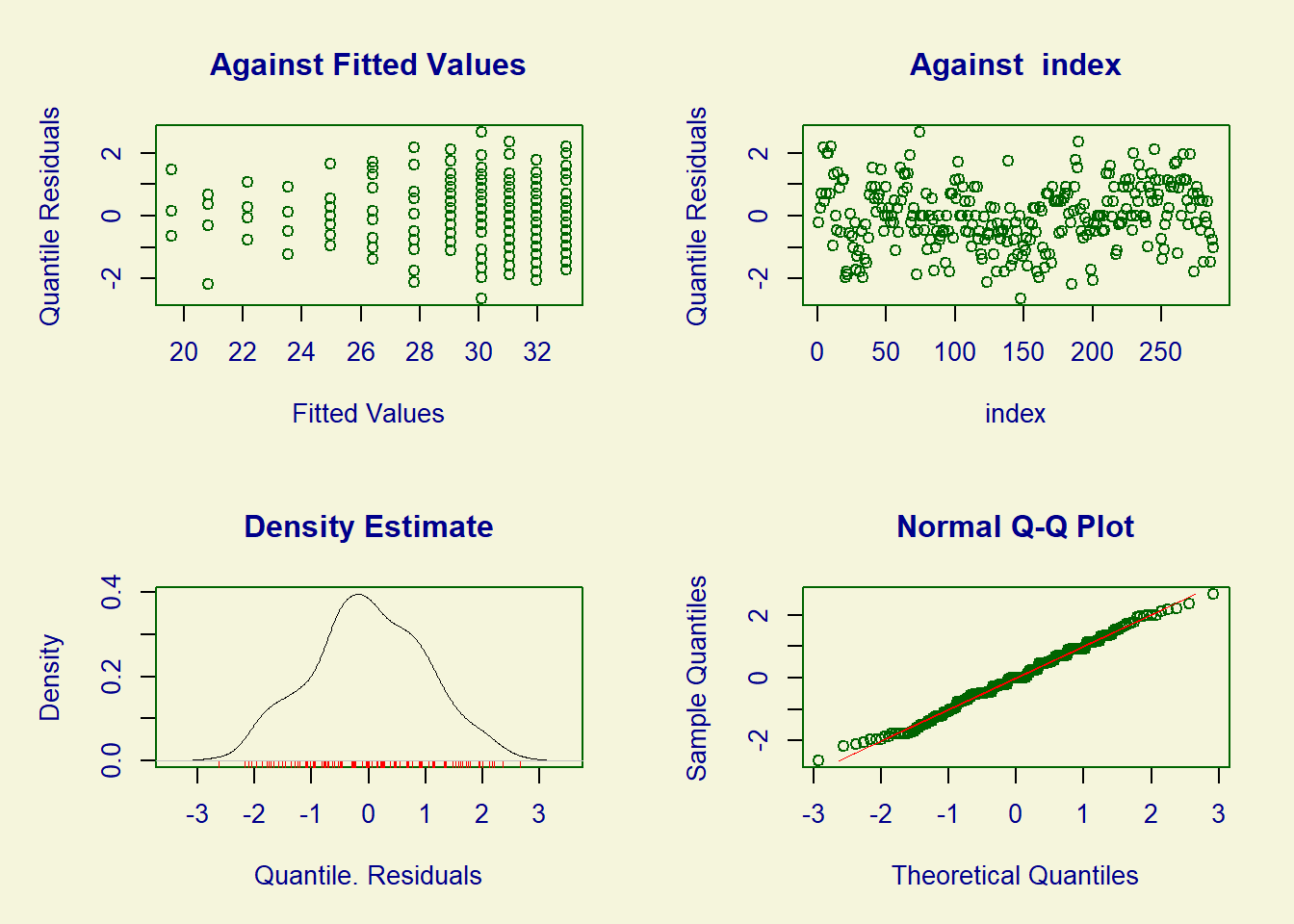


## ******************************************************************

## Summary of the Quantile Residuals

## mean = 0.0001419776

## variance = 1.003485

## coef. of skewness = -0.0006356002

## coef. of kurtosis = 2.619106

## Filliben correlation coefficient = 0.9969149

## ******************************************************************

wp(m0)


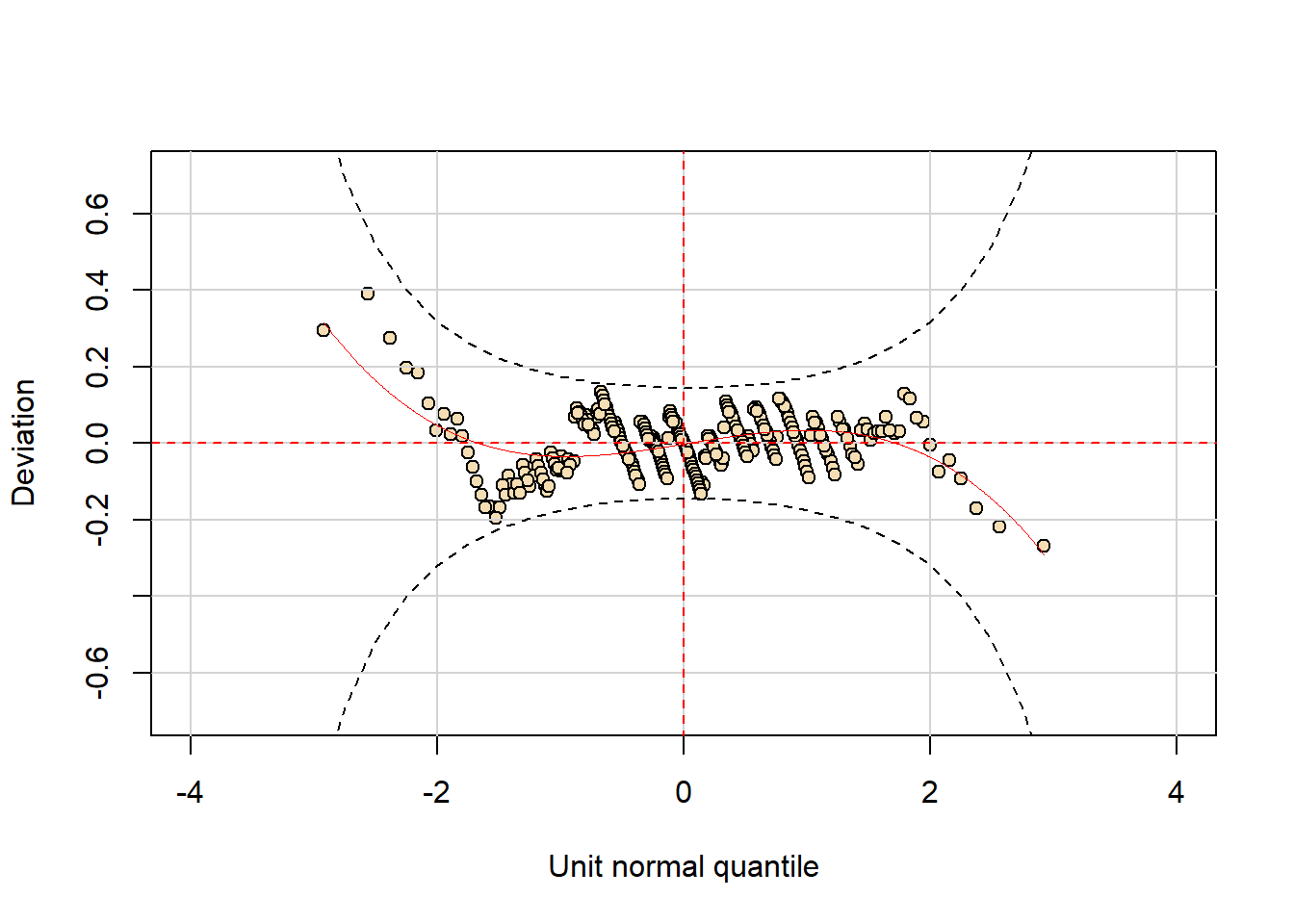


centiles(m0,bd$IGCsem)


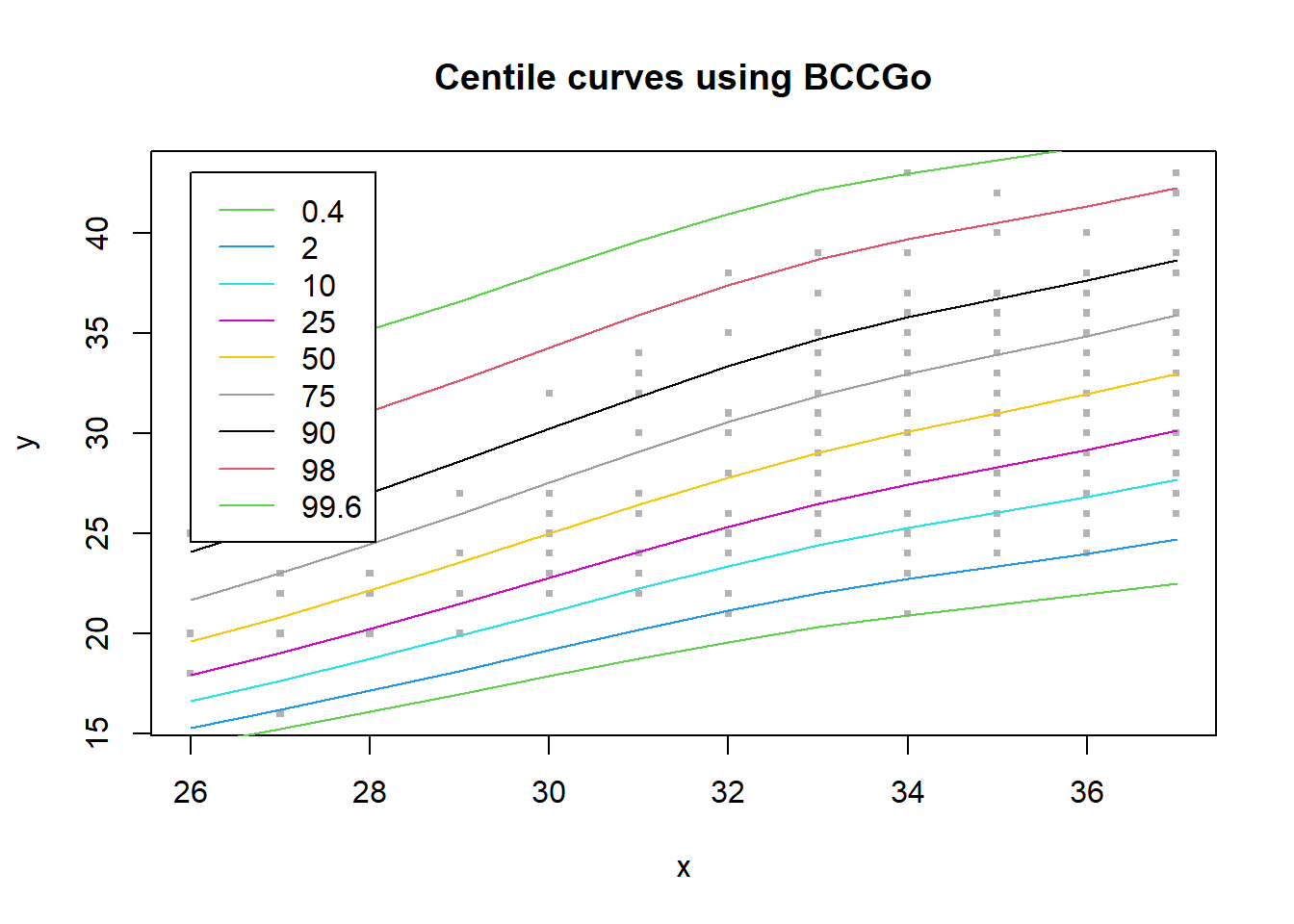


## % of cases below 0.4 centile is 0

## % of cases below 2 centile is 1.388889

## % of cases below 10 centile is 11.45833

## % of cases below 25 centile is 23.61111

## % of cases below 50 centile is 49.65278

## % of cases below 75 centile is 71.875

## % of cases below 90 centile is 89.23611

## % of cases below 98 centile is 98.26389

## % of cases below 99.6 centile is 99.65278

centiles.fan(m0,bd$IGCsem,cent=c(5,25,50,75,95),colors = "cm")


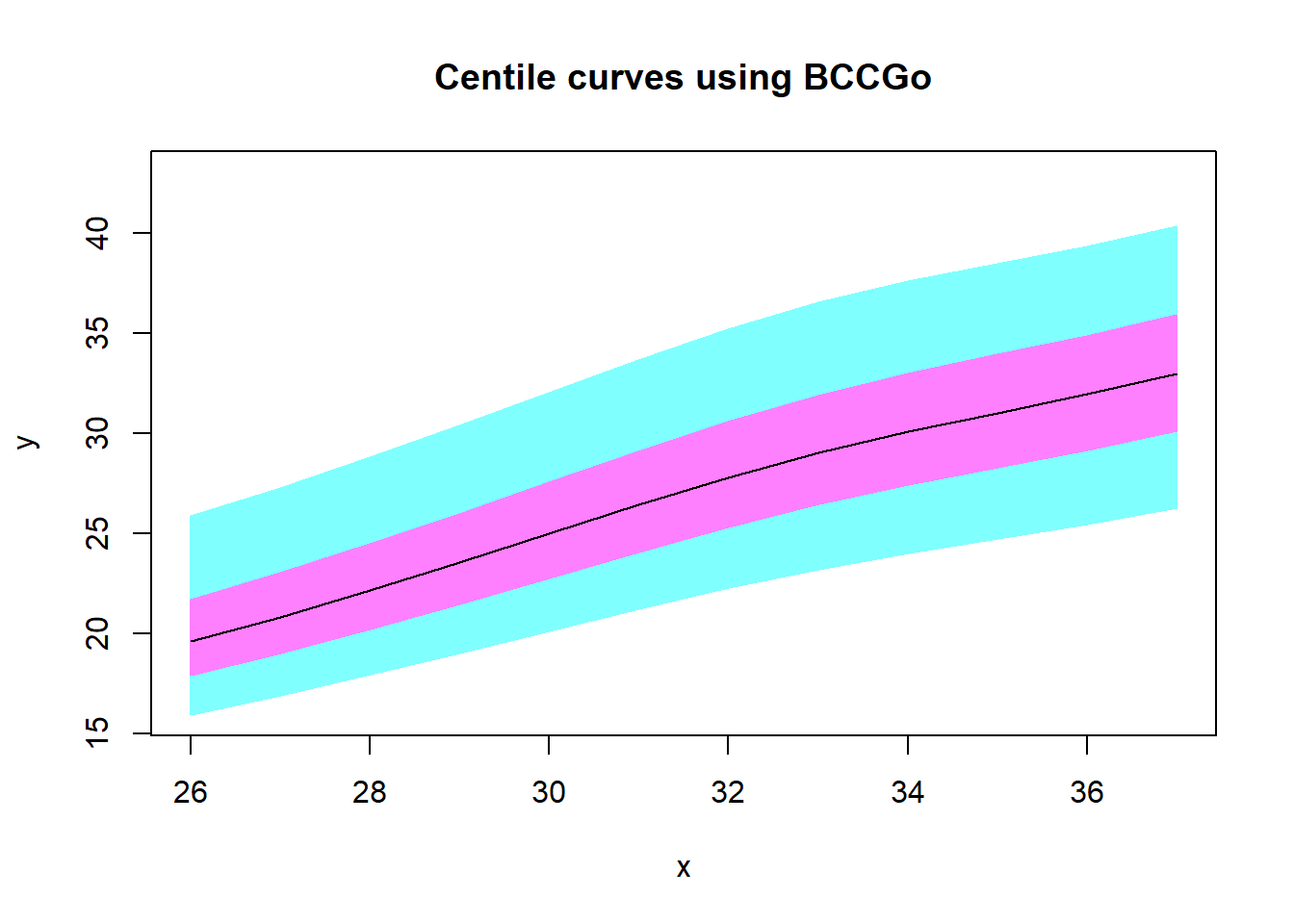


*#predizer percentis de CPT dada IGCsem*

IGCsem_exemplos=seq(26,37,1)

matriz_predicoes = centiles.pred(m0,xname="IGCsem",xvalues=IGCsem_exemplos,cent=c(5,25,50,75,95),plot=T)


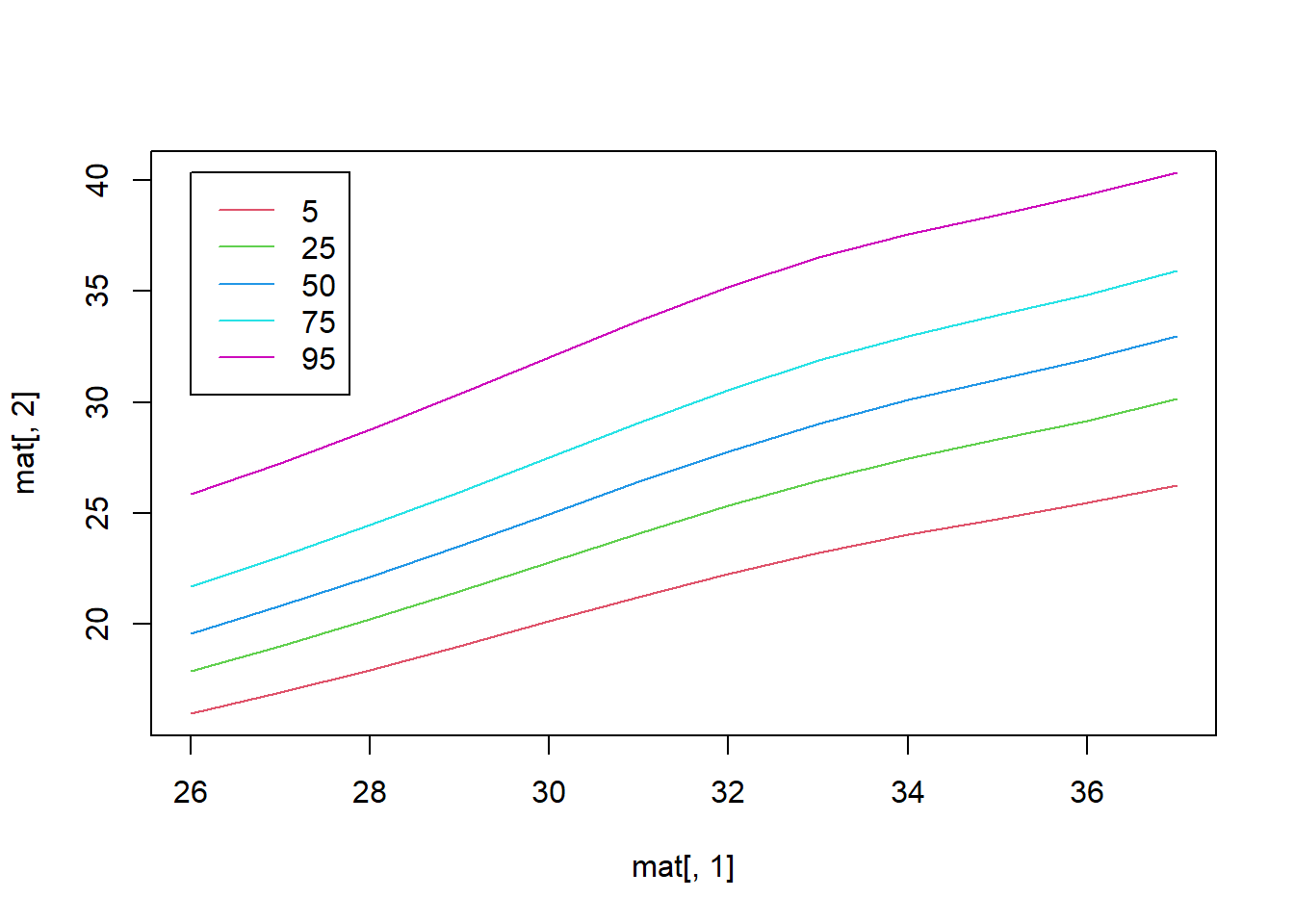


matriz_predicoes

## x 5 25 50 75 95

## 1 26 15.98123 17.90790 19.59863 21.70422 25.85885

## 2 27 16.94160 19.02892 20.83482 23.04636 27.27647

## 3 28 17.96017 20.22252 22.15154 24.47477 28.78982

## 4 29 19.02954 21.48110 23.54037 25.97967 30.38576

## 5 30 20.13085 22.78343 24.97736 27.53356 32.02985

## 6 31 21.22955 24.08880 26.41600 29.08298 33.65629

## 7 32 22.28572 25.34850 27.79996 30.56300 35.18646

## 8 33 23.24082 26.49234 29.04990 31.88489 36.51842

## 9 34 24.04930 27.46563 30.10487 32.98231 37.58063

## 10 35 24.75937 28.32240 31.02476 33.92328 38.45745

## 11 36 25.47724 29.18690 31.94856 34.86386 39.33343

## 12 37 26.27298 30.14322 32.97097 35.91021 40.33218

*#predizer escores z de CPT dada mesmas IGCsem*

matriz_predicoes_z = centiles.pred(m0,xname="IGCsem",xvalues=IGCsem_exemplos, type="standard-centiles",dev=c(-2.5,-2,-1,0,1,2,2.5),plot=T)


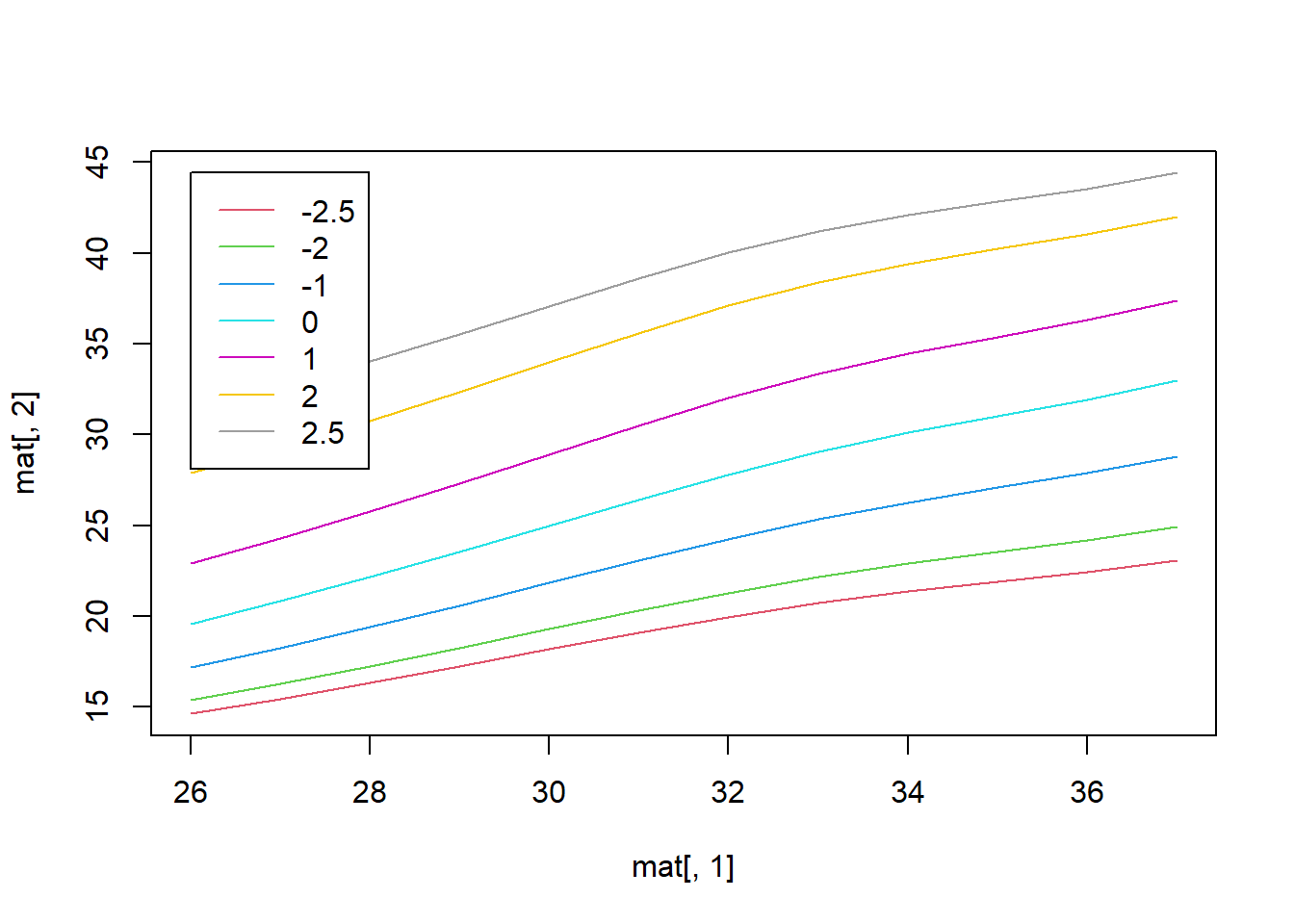


matriz_predicoes_z

## IGCsem -2.5 -2 -1 0 1 2 2.5

## 1 26 14.63278 15.38800 17.20460 19.59863 22.91945 27.88432 31.44901

## 2 27 15.46448 16.29327 18.27040 20.83482 24.30254 29.27070 32.66842

## 3 28 16.34036 17.25098 19.40426 22.15154 25.77421 30.76302 34.03294

## 4 29 17.25238 18.25355 20.59877 23.54037 27.32382 32.34525 35.51534

## 5 30 18.18248 19.28260 21.83370 24.97736 28.92187 33.97886 37.06629

## 6 31 19.09946 20.30515 23.07068 26.41600 30.51112 35.59215 38.60202

## 7 32 19.96887 21.28385 24.26426 27.79996 32.02226 37.10068 40.02756

## 8 33 20.74028 22.16377 25.34841 29.04990 33.36206 38.39758 41.22801

## 9 34 21.37510 22.90245 26.27156 30.10487 34.46208 39.41004 42.12857

## 10 35 21.91737 23.54634 27.08554 31.02476 35.39523 40.22958 42.83130

## 11 36 22.45919 24.19566 27.90819 31.94856 36.32662 41.05200 43.54788

## 12 37 23.05856 24.91552 28.81877 32.97097 37.36825 42.00710 44.41607

*#predizer escores z dados CPT e IGCsem de individuos*

centiles(m0,bd$IGCsem)


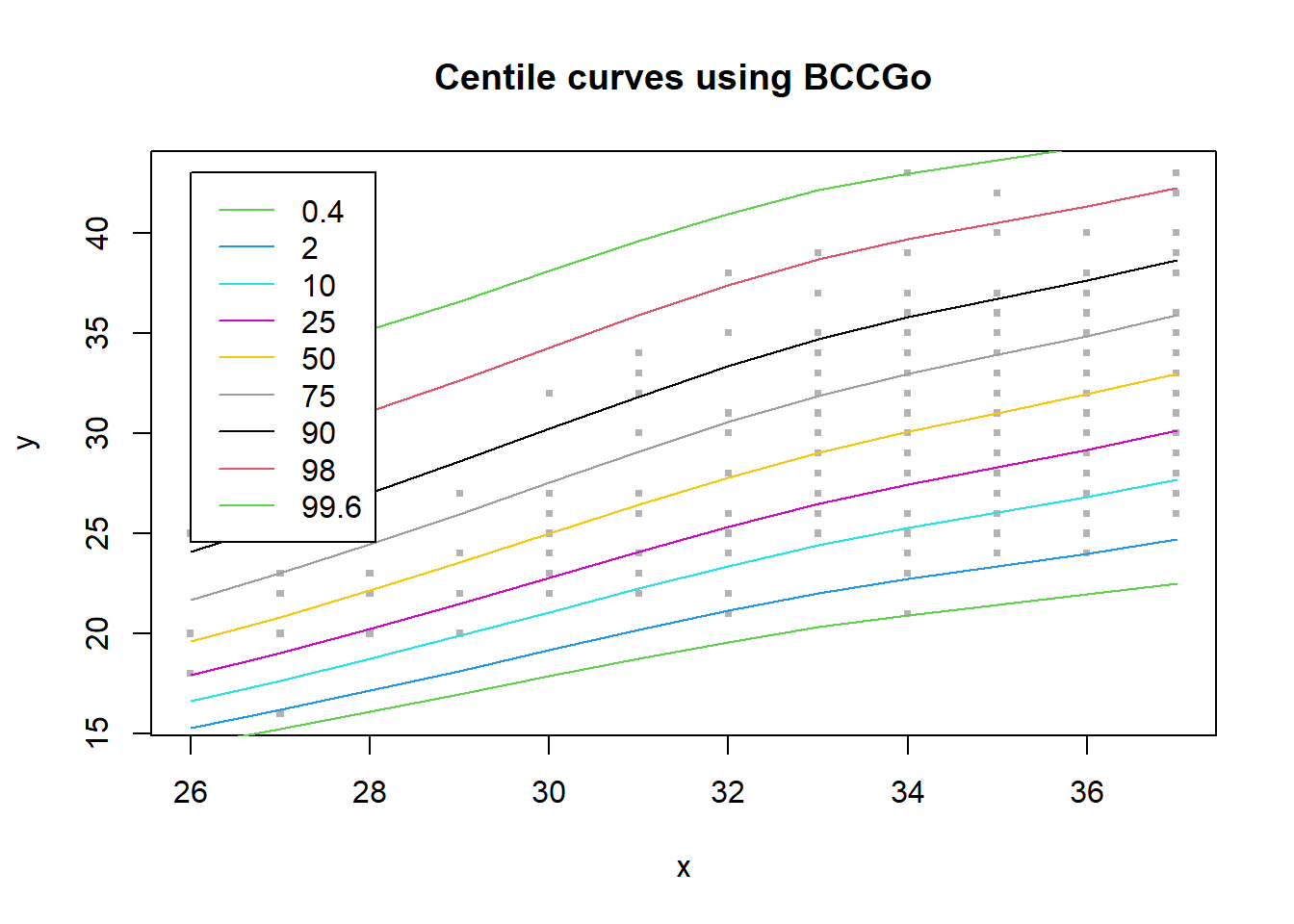


## % of cases below 0.4 centile is 0

## % of cases below 2 centile is 1.388889

## % of cases below 10 centile is 11.45833

## % of cases below 25 centile is 23.61111

## % of cases below 50 centile is 49.65278

## % of cases below 75 centile is 71.875

## % of cases below 90 centile is 89.23611

## % of cases below 98 centile is 98.26389

## % of cases below 99.6 centile is 99.65278

ind_IGCsem = c(30,34)

ind_CPT=c(33,25)

matriz_predicoes_z_ind = centiles.pred(m0,xname="IGCsem",xval=ind_IGCsem, yval=ind_CPT,type="z-scores")

matriz_predicoes_z_ind

## [1] 1.825865 -1.362369

**Supplementary material 2 – Bland-Altman Analyses**

**
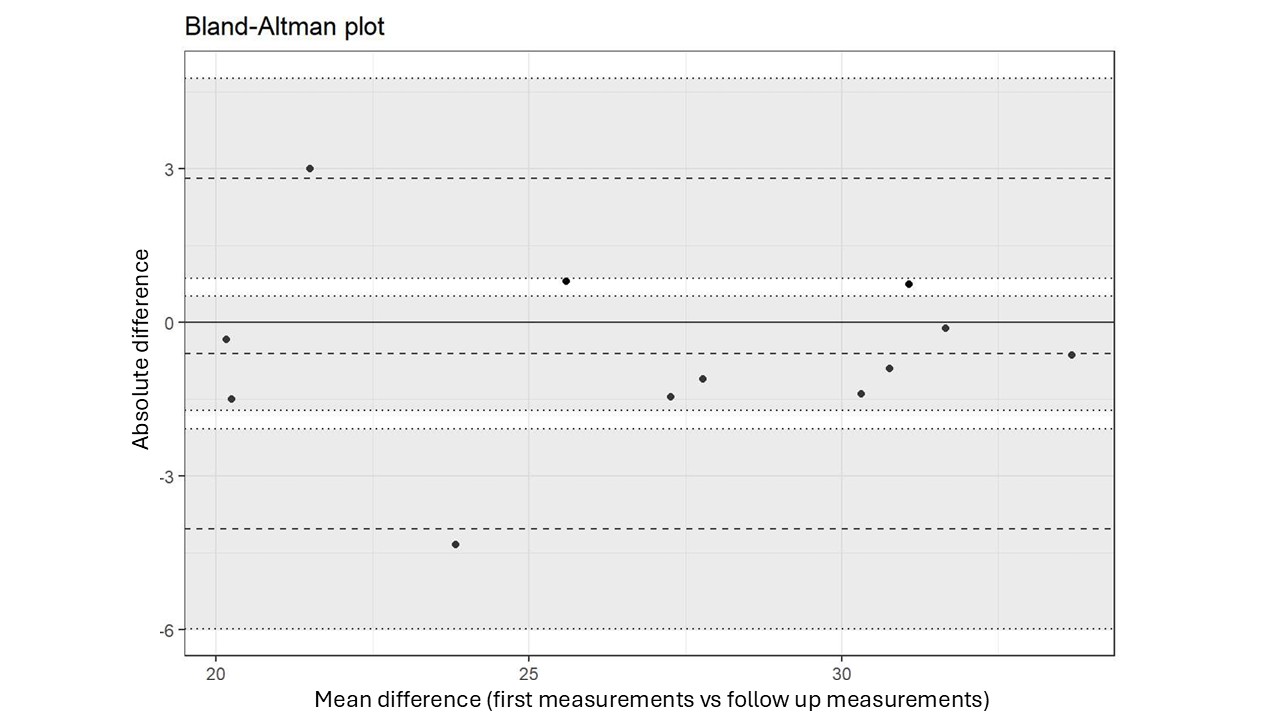
**

## Bland-Altman Statistics

## =======================

## t = -1.1971, df = 11, p-value = 0.2564

## alternative hypothesis: true bias is not equal to 0

##

## =======================

## Number of comparisons: 12

## Maximum value for average measures: 33.68

## Minimum value for average measures: 20.16667

## Maximum value for difference in measures: 3

## Minimum value for difference in measures: -4.333333

##

## Bias: -0.6030167

## Standard deviation of bias: 1.744993

##

## Standard error of bias: 0.5037362

## Standard error for limits of agreement: 0.8862524

##

## Bias: -0.6030167

## Bias- upper 95% CI: 0.5056992

## Bias- lower 95% CI: -1.711733

##

## Upper limit of agreement: 2.81717

## Upper LOA- upper 95% CI: 4.767798

## Upper LOA- lower 95% CI: 0.8665419

##

## Lower limit of agreement: -4.023204

## Lower LOA- upper 95% CI: -2.072575

## Lower LOA- lower 95% CI: -5.973832

##

## =======================

## Derived measures:

## Mean of differences/means: -2.240634

## Point estimate of bias as proportion of lowest average: -2.990165

## Point estimate of bias as proportion of highest average -1.79043

## Spread of data between lower and upper LoAs: 6.840374

## Bias as proportion of LoA spread: -8.815551

##

## =======================

## Bias:

## -0.6030167 ( -1.711733 to 0.5056992 )

## ULoA:

## 2.81717 ( 0.8665419 to 4.767798 )

## LLoA:

## -4.023204 ( -5.973832 to -2.072575 )
